# Sources of attitudes towards parent–child co‐sleeping and their effects: A systematic scoping review

**DOI:** 10.1111/famp.13022

**Published:** 2024-06-04

**Authors:** Sarah P. Kruse, Levita D'Souza, Hannah G. G. Tuncer, Sandra E. Stewart

**Affiliations:** ^1^ Faculty of Education Monash University Melbourne Victoria Australia

**Keywords:** attitudes, bed‐sharing, co‐sleeping, room‐sharing, systematic scoping review

## Abstract

Parent–child co‐sleeping is a common practice in many cultures, although in Western countries, families who engage in parent–child co‐sleeping can encounter attitudes about co‐sleeping that feel critical from the people around them, as it is not commonly accepted and often stigmatized. This systematic scoping review examined and synthesized the available literature on the attitudes about parent–child co‐sleeping that people encounter, their origins, and their effect on parents' own attitudes and behaviors. A total of 9796 abstracts were screened, and 33 studies were included. While the scope of the literature on this topic was narrow, this review demonstrated that parents/caregivers mostly encounter encouraging attitudes about co‐sleeping from their extended family members and within their culture and discouraging attitudes from healthcare professionals. Findings suggest that encouraging attitudes enhance the likelihood of parents engaging and continuing with co‐sleeping behavior, while discouraging attitudes can lead to the avoidance of parents discussing sleep with their healthcare professionals and can cause conflicts with other family members, including partners. Based on these findings, we conclude that further research is needed in several areas related to co‐sleeping in Western culture, most specifically in how external attitudes influence the decision to co‐sleep, as well as other behaviors and cognitions such as engagement with healthcare professionals, family satisfaction, parental self‐efficacy, and overall mental health.

## INTRODUCTION

Parent–child co‐sleeping is when a parent and child sleep in close proximity to each other (Goldberg & Keller, 2007; McKenna et al., 2007). Distinctions are made between bed‐sharing (sharing the same sleeping surface) and room‐sharing (sharing the same room, but not the same sleeping surface), while the umbrella term co‐sleeping refers to shared sleeping, including bed‐sharing, room‐sharing, and other proximity sleeping arrangements (McKenna et al., 2007; Mileva‐Seitz et al., 2017). In this review, the umbrella term is used, referring to the practice of a parent or caregiver sleeping within close proximity to an infant or child, including room‐sharing, surface‐sharing, and the use of co‐sleeping aids in close proximity to a sleeping caregiver (Goldberg & Keller, 2007; McKenna et al., 2007).

From a biological evolutionary perspective, co‐sleeping is essential for child protection and development (Barry, 2019; Mileva‐Seitz et al., 2017) and beneficial in encouraging parent–child bonding, and promoting secure attachment into toddlerhood (Barry, 2019; Bowlby, 1973; Higley & Dozier, 2009). Current literature examining reasons for co‐sleeping has found that co‐sleeping aids with breastfeeding (D'Souza et al., 2023; Goldberg & Keller, 2007; Salm Ward, 2015), preserving maternal sleep due to the reduced frequency of getting out of bed (Salm Ward, 2015), and that co‐sleeping assures children of attention, care, and security (McKenna et al., 2007; Thoman, 2006). Given this, co‐sleeping has been strongly embedded in culture and was historically the default sleeping practice (McKenna et al., 2007).

Literature has also examined why parents begin co‐sleeping, distinguishing between proactive (co‐sleeping as a conscious choice) and reactive (intended on solitary sleeping but co‐slept in reaction to other factors) co‐sleeping, finding that intentionality can influence perceptions on how co‐sleeping affects maternal and child sleep (Keller & Goldberg, 2004; McKenna & Volpe, 2007; Mileva‐Seitz et al., 2017; Ramos et al., 2007).

There are also considerable cross‐cultural differences in co‐sleeping (Andre et al., 2021; Mileva‐Seitz et al., 2017; Mindell et al., 2013). In Eastern countries (e.g., India, and Japan), co‐sleeping continues to be a common practice (Mindell et al., 2010; Owens, 2004; Shimizu et al., 2014), with the prevalence relatively stable across generations (Green & Smith, 2007; Owens, 2002), while in Western countries, co‐sleeping is comparatively lower during infancy, toddlerhood, and childhood (Mileva‐Seitz et al., 2017; Mindell et al., 2013), although there are variations seen across cultural groups and races (Colson et al., 2013; Salm Ward, 2015). Prevalence rates of co‐sleeping can also be impacted by what methods and classifications are used to collect such data (Ball et al., 1999).

Since industrialization in Western countries, there has been a shift in the acceptance of co‐sleeping due to a converging change in politics, economic climates, and social values, with independence becoming heavily valued (McKenna et al., 2007; Mileva‐Seitz et al., 2017; Thoman, 2006). The belief that solitary sleep promotes child independence, a shift towards privacy, and concerns with health and disease, reinforced the message of solitary sleep as superior (Andre et al., 2021; McKenna et al., 2007; Owens, 2004). Safety concerns regarding the risk of sudden unexpected death in infancy (SUDI) and sudden infant death syndrome (SIDS) have also been expressed about co‐sleeping, specifically bed‐sharing (Carpenter et al., 2013; Hoffend & Sperhake, 2014), although it has been suggested that many of these claims have methodological shortcomings such as failure to account for other risk factors (e.g., low birth weight, the use of alcohol or drugs) (Blair et al., 2014; Gessner & Porter, 2006; McKenna et al., 2007; Mileva‐Seitz et al., 2017). Furthermore, terminology inconsistencies, such as using SIDS and SUDI interchangeably and including potentially hazardous practices (e.g., sofa‐sharing) under the same category, have made results difficult to interpret (Goldberg & Keller, 2007; McKenna et al., 2007; Ottaviani, 2016). Additionally, with the high value on independence and autonomy, there are concerns that co‐sleeping will stunt independence development, resulting in family stress and dissatisfaction (Ahlborg & Strandmark, 2001).

Most research aiming to challenge negative perceptions of co‐sleeping concludes with recommendations for healthcare professionals to examine co‐sleeping families holistically and to provide education on safe co‐sleeping, taking a risk minimization approach (D'Souza & Cassels, 2023; McKenna & Volpe, 2007; Owens, 2002; Thoman, 2006), allowing parents to discuss sleep without judgment. However, this is often not the most common practice, and parents who do co‐sleep face confusion and criticism (Ball, 2017; Countermine & Teti, 2010; Shimizu & Teti, 2018).

Parents encounter different attitudes about co‐sleeping from different sources, including midwives, nurses, and friends (Bailey, 2016; Shimizu & Teti, 2018), but overall, this information is limited. Other research reported that parents seek information from relatives, friends, books, magazines, physicians, and online forums about their child's sleep, but not specific to co‐sleeping (Johnson, 1991; Ramos, 2003). Literature regarding the sources of attitudes around co‐sleeping is scarce, with much of the focus being on general sleeping or parenting.

Furthermore, how these attitudes affect parents' own attitudes and behaviors is unknown. In their study, Bailey (2016) found that conflicts between advice from professionals and cultural norms made parents feel judged to the point where they avoided discussing co‐sleeping with medical professionals. Such experiences were also reported in a review by Salm Ward (2015), further highlighting that perceived judgment around co‐sleeping may result in apprehension to engage with healthcare services. This disengagement can be severely detrimental to the health of the family, as parents may engage in unsafe practices or generally avoid engaging with medical professionals for fear of being further scrutinized. A further consequence of judgment about co‐sleeping may be withdrawal from friends and family (Bailey, 2016; Neoh et al., 2022; Salm Ward, 2015). Such avoidance can lead to social isolation or avoidance of support seeking. While these studies highlight the potential impacts that stigmatism can have on parents, the full effect is relatively unknown.

### Present review

To the authors' knowledge, there is currently no review that has examined the literature on the co‐sleeping attitudes encountered by parents and their effects on their cognitions and behaviors. The aim of this review was to examine and synthesize the available literature on the attitudes about parent–child co‐sleeping that people encounter. Furthermore, the scope in which co‐sleeping attitudes are currently studied and reported in academic literature was reviewed.

## METHOD

A systematic scoping review was selected as the most appropriate method for this review, recognizing the objective was to examine the literature and identify gaps rather than answer questions or evaluate interventions (Arksey & O'Malley, 2005; Davis et al., 2009; Peters et al., 2015, 2020). Structural guidance was provided by the Preferred Reporting Items for Systematic Reviews and Meta‐Analyses Extension for Scoping Reviews checklist (PRISMA‐ScR) (Tricco et al., 2018) and the Joanna Briggs International (JBI) manual for evidence synthesis (Peters et al., 2020), allowing for transparency and rigor.

A systematic search on eight databases was conducted on April 7, 2022: CINAHL Plus, MIDIRS, MEDLINE (via Ovid), PubMed, Scopus, PsycInfo, Web of Science (core collection), and Cochrane Database of Systematic Reviews. Search terms are shown in Table 1.

**TABLE 1:** General search terms by concept.

Concept	Search terms
Co‐Sleeping	Co‐sleep* OR cosleep* OR bed‐shar* OR room shar* OR bed arrangement* OR sleep arrangement*
Attitudes and methods	attitude* OR percept* OR experience* OR perspect* OR voice OR advice OR view* OR surve* OR interview OR qualitative* OR quantitative* OR focus*
Subjects	child* OR parent*OR caregive* OR infan* OR adolesce* OR toddler* OR babies OR newborn* OR teen* OR nurse* OR healthcare OR professional* OR doctor* OR midwife* OR paediatr* or community* OR matern* OR mother* OR patern* OR father*

*Note*: An asterisk (*) was applied to these search terms in order to return words with different endings (e.g., child* would result in *child*, *children* and *childhood* being included in the search).

### Data screening

After generating an initial list of *n* = 12,150 studies, duplicates were removed (*n* = 2355). The remaining studies (*n* = 9795) were imported into Covidence and double‐screened by two authors, SK and HT, based on their title and abstract, followed by a full‐text screen. At this stage, the inclusion criteria were broad, allowing for reviewers to explore the scope and then define the criteria further if necessary. Studies had to (a) be written in English, (b) be from peer‐reviewed, primary research, (c) focus on parent–child co‐sleeping, and (d) include attitudes on parent–child co‐sleeping. No restrictions were put on the year of publication or country/cultures examined. Qualitative, quantitative, and mixed‐method study designs were all included.

Studies were excluded if they only focused on the prevalence rates or reported behavioral practice of co‐sleeping, without exploration of the attitudes driving these figures or when the link between outcomes and co‐sleeping behaviors was inferred by the studies' authors (rather than participants).

Review articles that were published between 2013 and 2022 and otherwise met the inclusion criteria were flagged, and a backward citation search was performed to check if they met the criteria for inclusion. From this process, *n* = 2 studies were included in the review.

Reviewers routinely met to discuss questions that arose regarding the criteria. Disagreements were resolved through discussion and taken to the wider team if they could not be resolved.

**FIGURE 1:**
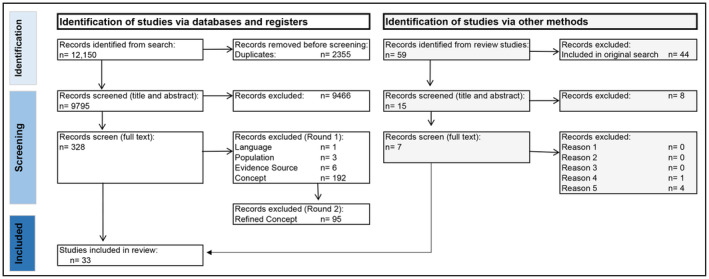
PRIMSA flowchart.

A total of 328 studies were included in the full‐text review. Following this, given the high number of articles suitable for extraction (*n* = 126), reviewers further refined the inclusion criteria to capture a more explicit focus, more closely aligned with the original research question, which aimed to focus on an examination of the sources of attitudes. Articles were double‐screened with an additional inclusion criterion: (e) studies must include where the participants' attitudes or beliefs around co‐sleeping have emerged from. At the conclusion of this screening, *n* = 31 studies were included in the review. The flow of records is shown in the PRISMA diagram (Figure 1), and a list of included studies can be found in Table S1.

While there is currently no consensus on the necessity to appraise the quality of the studies in a scoping review (Arksey & O'Malley, 2005), a quality evaluation was completed by reviewers HT and SK using JBI's Critical Appraisal Checklists (Lockwood et al., 2015; Moola et al., 2020). All articles were found to be of reasonably high quality (Table S2).

### Data extraction and synthesis

Extracted data were determined based on the aim of the review. The following data was extracted: general study information (i.e., year of publication, aim), participant demographics, definition of co‐sleeping, whether co‐sleeping was intentional or reactive, where and from whom attitudes were encountered, what the attitudes were, and the effect that these encounters had on other attitudes and behaviors.

To provide an overview of the scope of the data, the general characteristics of the studies were charted and described by frequency. The remaining data were charted, coded thematically, and synthesized using the constant comparative method, allowing researchers to find relations between categories and conceptualize the data further (Barnett‐Page & Thomas, 2009; Boeije, 2002).

## RESULTS

Thirty‐three studies met the inclusion criteria (See Table 2 for detailed study and participant characteristics). In summary, more than half (21/33, 63.6%) were published in the past 10 years; and almost all studies were conducted in Western countries (28/33, 84.8%). Most studies focused on the experiences of parents or caregivers (28/33, 84.8%), and most of these were on mothers' experiences (17/28, 60.7%). Other populations examined included pregnant women (Feld et al., 2021), lactation consultants (Hodges et al., 2018), and pediatricians (Schaeffer & Asnes, 2018). Two studies included mixed populations (e.g., healthcare workers and parents) (Gaydos et al., 2015; Sadler et al., 2020). When examining the age of the co‐sleeping child, most studies (20/33, 60.6%) focused on infants under 1 year of age exclusively.

**TABLE 2:** Study and participant characteristics.

Reference	Definition of co‐sleeping	Country of origin	Participants	Age of children	Cultural identify/race/ethnicity of participants
Abel et al. (2001)	Not defined, used minimally	New Zealand	Parents	Infants	Māori, Tongan, Samoan, Cook Islanders, Niuean & Pakeha
Abels et al. (2021)	Co‐sleeping includes both room‐sharing and bed‐sharing	Norway	Mothers	Infants	Norwegian
Bailey (2016)	Co‐sleeping includes “bed‐sharing, room sharing and sleeping with a child on a couch or sofa”	Australia	Mothers	Infants	Anglo‐Saxon & Asian
Caraballo et al. (2016)	Not defined, not used	USA	Teenage mothers	Infants & toddlers (2–21 months)	Hispanic or Latino, White, African American & ‘mixed’
Chianese et al. (2009)	Not defined, not used	USA	Caregivers	Infants (up to 6 months)	African American, White & ‘missing’
Crane and Ball (2016)	Not defined, not used	UK	Mothers	Infants (8–12 weeks old)	White British & Pakistani
Dodd and Jackiewicz (2015)	Co‐sleeping defined as bed‐sharing, “as either one or both parents being asleep on the same sleeping space as the baby. This may include bed‐sharing or sleeping on a couch”	Australia	Mothers & grandmothers	Infants (2–12 months)	Non‐Aboriginal & Aboriginal
Dosanjh and Ghuman (1996)	Not defined, term used and appears to be referring to bed‐sharing	UK and Punjab, India	Mothers	N/A	Punjabi
Feld et al. (2021)	Not defined, not used	Ecuador	Pregnant women	Infants	Mestizo, Afro‐Ecuadorian, White, Montubio & Indigenous
Gaydos et al. (2015)	Not defined, not used	USA	Mothers & Medical Providers (data extracted from Providers)	Infants (up to 6 months)	Mothers: African American Providers: N/A
Gustafsson et al. (2022)	Not defined, term used and ambiguity in what it refers to explicitly	Europe	Parents	Infants & toddlers (0–3 years)	Swedish, Scandinavia & Non‐European
Hatton and Gardani (2018)	Defines co‐sleeping as, “a parent and child sleeping in close proximity and can be separated into room sharing, for example, when a parent and child sleep in the same area but on different surfaces, and bed‐sharing, for example when a parent and child sleep on the same bed”	UK	Mothers	Infants, toddlers & children (0 to 12 years)	White British & ‘mixed’
Hauck et al. (2008)	Not defined, term used minimally	USA	Mothers	Infants (up to 12 months)	White, Black, Hispanic & ‘other’
Herman et al. (2015)	Not defined, not used	USA	Mothers & supporters of mothers (e.g., partners, grandmothers)	Infants & toddlers (up to 2 years)	American Indian/Alaskan Native, Black or African American & Other (Hispanic or Caucasian)
Hirai et al. (2019)	Not defined, not used	USA	Mothers	Infants	Non‐Hispanic White, Non‐Hispanic Black, Hispanic, Non‐Hispanic American Indian or Alaska Native, Non‐Hispanic Asian or Pacific Islander & Non‐Hispanic multiple races
Hodges et al. (2018)	Not defined, used minimally	USA	Lactation Consultants	Infants	Caucasian & African American
Hooker et al. (2001)	Co‐sleeping defined as, “infants and parents sleeping together on the same surface”	UK	Mothers & fathers	Infants	N/A
Jones et al. (2017)	Co‐sleeping defined as, “separate sleep surface but close proximity”	New Zealand	Parents	Infants & toddlers (2 months to 2 years)	Māori
Lapps Wert et al. (2015)	Co‐sleeping defined as, “an infant sleeping close to his parents,” and noting bed‐sharing is a subset of co‐sleeping, “where an infant is put to sleep in the parent's bed with them”	USA	Mothers	Infants & toddlers (6 months to 2 years)	Caucasian, African American & Latina
Liamputtong (2002)	Not defined, term used and appears to be referring to bed‐sharing	Australia	Mothers	Newborns	Hmong
MacFarlane et al. (2021)	Not defined, not used	New Zealand	Parents	Infants	Māori, Pasifika & European and Asian
Martinez and Thompson‐Lastad (2015)	Not defined, used minimally	USA	Parents	Toddlers & children (2 to 5 years)	Latino
McKenna and Volpe (2007)	Co‐sleeping defined as, “caregiver who sleeps within close enough proximity to the infant to permit the exchange of at least two sensory stimuli”	Canada, US, Australia, and UK	Mothers	Infants	N/A
Morelli et al. (1992)	Co‐sleeping defined as, “being in the mother's bed”	USA and Guatemala	Mothers	Infants & toddlers (up to 2 years)	USA (unspecified) & Guatemalan
Ramos (2003)	Co‐sleeping is defined as, “children and parent sleep at night in the same room, often in the same bed”	USA	Mothers	Infants, toddlers & children (6 months to 5 years)	White & and others not reported
Sadler et al. (2020)	Not defined, term used, appears to refer to bed‐sharing	USA	Parents & Healthcare and childcare providers (data extracted from parents)	Infants & toddlers	African American, White, Asian, Multiracial, Other & Unknown
Schaeffer and Asnes (2018)	Not defined, not used	USA	Pediatricians	Infants	N/A
Smith et al. (2010)	Not defined, not used	USA	Mothers	Infants (up to 8 months)	African American, Latino, White & Other
Specker et al. (2020)	Not defined, not used	USA	Mothers	Infants	White Non‐Hispanic, American Indian & Other races
Stiffler et al. (2016)	Not defined, term used, appears to be bed‐sharing	USA	Mothers of infants that died from SUDI	Infants	White, African American, Burmese & Mexican American
Stiffler et al. (2020)	Not defined, used minimally	USA	New mothers	Infants	African Americans
Tan et al. (2009)	Co‐sleeping defined as “sharing a bed with a parent”	USA and ‘other countries’	Parents	Infants & toddlers (up to 72 months)	Chinese (children) & White (parents)
Weimer et al. (2002)	Co‐sleeping defined as, “the presence of a child sleeping on the same mattress as an adult, within touching distance, for any length of time”	USA	Caregivers (Mothers 93%)	Infants, toddlers & children (up to 5 years)	African‐American & and others not reported

Most studies used qualitative methods (23/33, 69.7%), while seven (21.2%) used quantitative methods. Three studies (9.1%) used mixed methods, although the extractable data were from the qualitative component (Abels et al., 2021; Feld et al., 2021; Jones et al., 2017).

### Definitions of co‐sleeping

Table 2 provides further information on the definitions used. Twelve studies (36.4%) clearly defined the term co‐sleeping. Of these, seven studies (41.2%) referred to co‐sleeping as the child and parent/caregiver being in close proximity and including multiple sleeping arrangements (e.g., room‐sharing, bed‐sharing), while nine referred to co‐sleeping as bed‐sharing (52.9%), and one as room‐sharing without surface sharing (5.9%). Five studies (15.2%) used the term but did not provide an explicit definition. The remaining studies (16/33, 48.5%) did not define co‐sleeping but the term was either not used or used minimally, instead referring to other terms (e.g., bed‐sharing, room‐sharing). Further to this, the results extracted from most studies (28/33, 84.8%), specifically referred to attitudes about bed‐sharing. One study noted that most of its participants were bed‐sharing and therefore attitudes are in relation to this (Ramos, 2003), another examined room‐sharing without bed‐sharing (Hirai et al., 2019), and another reported results for bed‐sharing and room‐sharing separately (Specker et al., 2020). Two studies were more ambiguous, either combining room‐sharing and bed‐sharing results that were extracted for the review (Martinz & Thompson‐Lastad, 2015), or generally the results included in the review weren't explicit in what they were referring to (Abels et al., 2021). Only four studies discussed the distinction between proactive and reactive co‐sleepers.

### Sources of attitudes

Two groups were examined: ‘parents/caregivers’ (29/33, 87.9%) and ‘healthcare professionals’ (2/33, 6.1%). Additionally, while two studies used both healthcare professionals and parents/caregivers as participants, data could only be extracted for one population in each study (parent data only [Sadler et al., 2020]; healthcare professional data only [Gaydos et al., 2015]).

Table S2 provides an overview of the sources and attitudes encountered by parent/caregivers and their effects. Parents/caregivers described encountering attitudes about parent–child co‐sleeping from seven sources: healthcare professionals (e.g., pediatricians, midwives, 18/30, 60.0%), extended family members (e.g., parents, grandparents, 12/30, 40.0%), culture and traditions (11/30, 36.7%), friends (6/30, 20.0%), partners (4/30, 13.3%), literature (1/30, 3.3%), and media (1/30, 3.3%). There was an overlap seen between reports of family members and culture, with parents/caregivers noting the fusion of the two sources.

The three articles that provided insight into the attitudes encountered by healthcare professionals found that the sources of co‐sleeping attitudes came from mothers, parents generally, and from an employer (e.g., governing body such as the American Academy of Pediatrics).

### Discouraging and encouraging attitudes

A total of 91 attitudes from the included papers were analyzed. More discouraging attitudes were encountered by parents/caregivers towards co‐sleeping in the examined literature than encouraging attitudes (43/91, 47.3% vs. 41/91, 45.1%). Other attitudes were neutral or salient, with no direct expression towards encouraging or discouraging co‐sleeping (e.g., you can choose to co‐sleep or not) (6/91, 6.6%). One attitude encouraged room‐sharing while discouraging bed‐sharing explicitly (Hirai et al., 2019). Most bed‐sharing specific attitudes (*n* = 83) were also discouraging (41/83, 48.2%, 37/83, 44.6% encouraging, and 5/83, 6.2% neutral).

Discouraging attitudes focused on the dangers of co‐sleeping (14/44, 31.8%), that co‐sleeping was an undesirable habit with developmental or social consequences (8/44, 18.2%), and that co‐sleeping should be avoided because it was not the norm (1/44, 2.3%). There were also occurrences in which the specific sentiment itself was not clearly specified (20/44, 45.5%), but the overall attitude was discouraging (e.g., the source said to avoid co‐sleeping but had not specified why). Discouraging attitudes that specified co‐sleeping as dangerous or risky came from healthcare professionals, while discussions about co‐sleeping being an undesirable habit were seen more from friends, family, or partners.

Encouraging attitudes were primarily focused on the normality of co‐sleeping (11/41, 26.8%), noting it stems from tradition, spirituality, or habit. Other encouraging attitudes towards co‐sleeping noted its protective properties (7/41, 17.1%), discussing that co‐sleeping made babies feel secure and assisted with bonding and breastfeeding. Other sources discussed both normalcy and protection (2/41, 4.9%), and a high portion was non‐specific (21/41, 51.2%) (e.g., generally endorsed co‐sleeping or approved of the practices). The most common sources of encouraging attitudes were culture and/or tradition (13/41, 31.7%) and extended family members (10/41, 24.4%).

When examining attitudes encountered by healthcare professionals, attitudes about co‐sleeping from parents and mothers were encouraging (e.g., co‐sleeping aids with breastfeeding, co‐sleeping is a cultural practice), while attitudes from their employer (e.g., healthcare organization) were discouraging (co‐sleeping is unsafe).

### Additional effects of attitudes and behaviors

An effect of the attitudes towards co‐sleeping was recorded alongside the attitude in *n* = 66 of attitudes (66.7%). Firstly, the effect of attitudes on the co‐sleeping *behavior* of parents/caregivers was examined (40/66, 60.6%). In most cases (20/40, 50.0%), the attitudes encouraged parents to co‐sleep or reinforced the co‐sleeping they were already engaged in. A high portion of parents/caregivers also noted that their behavior change (or reinforcement) was paired with a change in their own attitude towards co‐sleeping (*n* = 13), with most of these attitudes being that co‐sleeping was encouraged or positive (*n* = 12). Overall, most encouraging attitudes that had led to engagement in or reinforcement of co‐sleeping were encountered from culture (*n* = 8).

Contrastingly, 12 studies reported a discouraging effect of others' attitudes that led to parents avoiding, stopping, or delaying their engagement in co‐sleeping. Four occurrences of behavior change also reported attitude change towards co‐sleeping (i.e., feeling unsure, general negative feelings and specific concerns that co‐sleeping should be avoided due to it being a bad habit difficult to break). The attitudes within studies that led to disengagement from co‐sleeping mostly came from healthcare professionals (*n* = 6), partners (*n* = 3), friends (*n* = 2), and extended family (*n* = 1). Healthcare professionals often referred to the safety risk, while friends and partners more often discussed co‐sleeping as having undesirable social or developmental consequences. Finally, in eight studies, participants reported that the attitudes of others (mainly healthcare professionals [*n* = 6]) had no effect on their co‐sleeping behavior.

Other attitude or behavior changes not specific to co‐sleeping were also examined. Parents/caregivers in four studies reported feeling judged, unsupported, or under surveillance after encountering attitudes about co‐sleeping from healthcare professionals and extended family members. Feelings of anger, frustration, or worry were also reported by parents/caregivers in three studies on four different occasions, most of which were a result of discouraging attitudes about co‐sleeping from healthcare professionals and the media. When examining the behavioral effects of healthcare professionals, Hodges et al. (2018) reported that lactation consultants felt as if the attitudes from their employer affected their work, restricting them from educating women on how to safely co‐sleep.

There were seven reports of parents/caregivers concealing their co‐sleeping practices, avoiding discussions about sleep arrangements, or ignoring advice (healthcare professionals, *n* = 6, friends, *n* = 1). There were also occurrences in which parents reported that attitudes lead to conflict or confrontation. Stiffler et al. (2020), reported that this was the result of discouraging attitudes from healthcare professionals, but the conflict was with extended family members who held encouraging attitudes to co‐sleeping. Dodd and Jackiewicz (2015) reported several instances of attitudes from partners resulting in conflict.

In other studies (*n* = 7), parents/caregivers reported feeling confident and supported with their decision to co‐sleep if it was in line with encouraging attitudes within their culture and within the written information they received (specific details not provided).

## DISCUSSION

Overall, this review has found that while many studies discuss attitudes towards co‐sleeping, few examine where these attitudes come from and the effects of these attitudes. Those that did examine this showed that encouraging attitudes towards co‐sleeping mostly come from culture and extended family, while discouraging attitudes mostly come from healthcare professionals. Reported effects were varied, with encouraging attitudes reinforcing co‐sleeping behaviors, while discouraging attitudes negatively impacted social and professional engagement.

### Definitions of co‐sleeping

A variety of definitions around co‐sleeping were present within this review, with many studies either failing to define co‐sleeping or related terms clearly or using them interchangeably. These findings further highlight the concern when using sub‐terms that fall underneath the term of co‐sleeping (e.g., bed‐sharing, room‐sharing), which hold different meanings that can influence participants' responses. One example is Hatton and Gardani (2018), who defined co‐sleeping themselves but noted that they did not do this with their participants. Other studies often used co‐sleeping to refer to bed‐sharing, although this was not always the case. Particularly in relation to guidelines, the definition of co‐sleeping vs. bed‐sharing vs. room‐sharing is important to distinguish, as the use of interchanging definitions can cause further confusion for parents.

While there was variety, and at times ambiguity in what co‐sleeping was defined as, most studies specified that the reported results were in relation to bed‐sharing, as expected given that most of the studies focused on co‐sleeping in infancy. Yet only a few studies outlined what encompassed bed‐sharing (e.g., waterbeds, sofas, co‐sleepers; Bailey, 2016; Chianese et al., 2009; Hauck et al., 2008). The review highlighted there were a high number of discouraging attitudes encountered, particularly from healthcare professionals in relation specifically to bed‐sharing, expressing concerns for the safety of the practice. Yet given bed‐sharing does occur for a variety of reasons (Barry & McKenna, 2022; Salm Ward, 2015) and is not always intentional (Cole et al., 2021; Ramos, 2003), a better approach may be to discuss how to bed‐share safely by avoiding circumstances that may increase risk (e.g., sleeping on sofas, when drugs or alcohol are involved; Blair et al., 2014; Gettler & McKenna, 2010) rather than completely discouraging caregivers (Blair, 2010; McKenna & McDade, 2005). When caregivers seek advice from healthcare professionals, ambiguity in definitions around these terms can misconstrue what is being spoken about, resulting in incorrect attitudes or advice being provided, possibly leading parents to feel judged. This review also highlights the lack of studies that focused on room‐sharing specifically, which may be protective against SIDS (McKenna & McDade, 2005).

In addition, most studies did not examine co‐sleepers based on their intentionality (proactive vs. reactive). As the intentionality of co‐sleeping can influence parents' perceptions of how co‐sleeping affects maternal and child sleep, and overall parental satisfaction (McKenna & Volpe, 2007; Ramos, 2003; Ramos et al., 2007), these results further suggest that much of the literature is missing a significant variable that can bias results (Kim et al., 2017; Mileva‐Seitz et al., 2017; Ramos, 2003). While this highlights the variation and inconsistencies in terminology, this was a minor feature of this review, and further examination is recommended.

### Culture, family, and healthcare professionals

In this review, participants' culture (that is an individual's background, racial or ethnic‐based beliefs and customs, rather than the residing country's culture) and extended family were the most frequently encountered sources of attitude reported, with these sources often intertwined.

Attitudes about co‐sleeping from extended family and culture were overwhelmingly more encouraging than discouraging. Within these studies, participants were often identified as belonging to a specific cultural identity, race, or ethnicity, such as Hmong living in Australia (Liamputtong, 2002), indigenous Punjabi (Dosanjh & Ghuman, 1996), or Indigenous populations (Dodd & Jackiewicz, 2015; MacFarlane et al., 2021). This noticeable, frequent discussion about encountering attitudes about co‐sleeping from these sources is not unexpected given previous literature (Colson et al., 2013; Desmosthesous & Desmosthesous, 2011; Jones et al., 2017; McKenna et al., 2007), and further highlights the inter‐relatedness of cultural and ethnic identity, and co‐sleeping.

The review also highlights that discouraging attitudes could be encountered from extended family members, with concerns relating to child development and social implications being mentioned, although not as frequently as concerns regarding risk from healthcare professionals. Given most of the studies originated from Western countries, many participants would have been likely to encounter Western societal values around sleep based on individualism and privacy, possibly accounting for this result (Andre et al., 2021; McKenna et al., 2007; Owens, 2004). While some studies examined participants' culture or ethnicity, for most, this was secondary, and the data limited. Given these few results and the overall prevalence of co‐sleeping within particular countries, cultures, and ethnicity groups, this review suggests that parents/caregivers who come from a cultural background that views co‐sleeping as culturally normal and acceptable may be more likely to encounter encouraging attitudes about the practice from extended family and within their culture compared to parents/caregivers from cultures (or with family members from cultures) who do not view co‐sleeping as normal.

There is limited literature on the extraneous effects of attitudes from culture and extended family. There were a few mentions of what might occur, for example, in Jones et al. (2017), it was reported that disillusionment from one's culture's views resulted in parents feeling unsupported and negatively judged by family, while Chianese et al. (2009) reported participants had feelings of confidence in their parenting after encountering encouraging attitudes from their culture. According to Dressler (2012)'s theory of cultural consonance, the cohesion or discord between individual's beliefs and behaviors, and that of the residing cultures, can affect one's psychological and physical stress. However, in such cases, the identification of said culture (or the societal expectations and values of the country in which they reside) also needs to be examined. The influence of cultural behaviors, and attitudes may be dependent on how aligned or misaligned their behaviors and beliefs are with that culture, and whether the culture is socially or familiarly grounded.

Within this, reflection on how societal expectations and values that shape attitudes towards co‐sleeping can then be passed to caregivers through healthcare professionals is considered. Barry (2019) and Ball (2006) discuss how in the U.S., advice from healthcare professionals stems not only from risk concerns but also from the values and expectations of society (e.g., early independence). Such expectations may not be considerate of familial and culture values or psychosocial factors. Here, discussions regarding racial inequality and healthcare in Western cultures should be considered. Research, and by extension advice, around infant safer sleep and co‐sleeping frequently point to risk factors such as alcohol and drug use (Blair et al., 2014) to be avoided, while favoring room‐sharing but not bed‐sharing in infancy, and solitary sleeping in separate rooms in toddler‐ and childhood. Wording here is often absolute (e.g., should/should not, do/do not), with the focus of risk‐elimination central to these guidelines and strategies (Harrison, 2022; Shatz & Blair, 2023). Yet in making such finite statements, consideration is not being made to marginalized families, who due to a multitude of factors such as education, mental health, and intergenerational trauma, may not be able to avoid such behaviors or circumstances. For example, in their study, Barajas and colleagues (2011) discuss racial disparities in bed‐sharing families, finding that African American and Hispanic families are more likely to co‐sleep with their toddlers in the U.S. compared to white, non‐Hispanic families. Following this, Chu and colleagues (2016) found that crowded housing conditions did influence bed‐sharing, their sample consisting mostly of African American mothers (58.8%), and discussed how this population, along with low socio‐economic status and education, faced an inhospitable housing market. Similar disparities were addressed by Dodd (2012) in Western Australia, reporting that there can be pragmatic reasons for co‐sleeping (e.g., limited access to materials such as cots and overcrowded living conditions) as well as cultural beliefs in the Australian Indigenous population, and that there was a need for culturally appropriate processes and information about co‐sleeping. Lack of access to resources may make changing generational patterns and circumstances less amenable to immediate change. Therefore, general sleep and co‐sleeping advice that does not acknowledge these disparities are, intentionally or otherwise, creating policies that favor one population over another and holding individuals responsible for infant mortality, rather than looking more broadly to the system (Braveman et al., 2022; Harrison, 2022). Initiatives such as the Pēpi‐Pod® Program (Grant et al., 2021; Tipene‐Leach et al., 2018; Young et al., 2018) aim to address this by exploring alternative approaches that focus on risk minimisation; however, overall, much more needs to be done in this space.

Ultimately, this review highlights how there appears to be little information on the interrelations between attitudes about co‐sleeping from one's environment, culture, and the people within these spaces (e.g., extended family, healthcare professionals), and how they ultimately affect parent/caregivers' other cognitions and behaviors.

Furthermore, as encouraging attitudes tended to promote co‐sleeping behavior, the review highlighted that parents/caregivers may avoid, stop, or be discouraged from co‐sleeping due to the discouraging attitudes that they encounter about co‐sleeping from other people, predominately from healthcare professionals (and specifically in relation to bed‐sharing). When examining other behavioral effects of these attitudes, the review showed examples in which parents concealed their co‐sleeping from healthcare professionals, with parents' choosing to do this for several reasons, including to avoid arguments, and in one case, out of fear for concerns that child protection services would be notified (Stiffler et al., 2020). Such avoidant behaviors can lead to negative outcomes, including parents not being given the opportunity to discuss how to co‐sleep safely (e.g., avoiding alcohol, no sofa sleeping) or general avoidance from healthcare professionals, which can lead to other negative outcomes such as post‐natal depression or early child‐developmental concerns being missed (Altfeld et al., 2017; Hunter, 2006; Owens, 2008).

### Self‐efficacy and mental health

This review also highlights that there were a large number of results that did not discuss if the attitude encountered by caregivers influenced their co‐sleeping behavior, other behaviors (e.g., avoidance of healthcare professionals), or other attitude changes (e.g., concerns about development), highlighting a gap in the literature.

Within Social Cognitive Theory sits the construct of self‐efficacy, the confidence in one's own ability to perform behaviors (Bandura, 1994). Research work on self‐efficacy and health outcomes has consistently found that low self‐efficacy is associated with lower mental health (Muris, 2002; Rabani Bavojdan et al., 2011), similarly with parental self‐efficacy and mental health (Cutrona & Troutman, 1986). Parental self‐efficacy refers to a parent's sense of capability to perform their role as a parent. Thus, when parents sense that they are underperforming, this can lead to negative outcomes such as poor mental health. One study comparing parenting self‐efficacy in co‐sleeping and non‐co‐sleeping parents did find that mothers who co‐slept reported significantly lower parenting self‐efficacy than non‐co‐sleepers, theorizing that if the co‐sleeping is undesired and therefore paired with anxiety and resistance, it may lead to mothers feeling less competent (Kim et al., 2017). It has also been suggested that when parents are comfortable with their care choices, they have feelings of increased self‐efficacy, which leads to other possible benefits such as improved mental health and better partner relationships (McDaniel & Teti, 2012; Thome & Skuladottir, 2005). The disconnect between parent's care choice and societal expectations that co‐sleeping parents in Western countries may encounter may then lead to feelings of judgment, resulting in low self‐efficacy and poorer mental health. This review aimed to provide insight into this by exploring whether studies examined how encountering different attitudes to a parent's own beliefs could affect their own parenting self‐efficacy, and as a result, their mental health. What was found was that these effects were minimally reported and have generally not explored within the literature.

### Partners

There was also a lack of partner attitudes identified in the review, with both discouraging and encouraging attitudes being reported (Bailey, 2016; Dodd & Jackiewicz, 2015; Herman et al., 2015; Ramos, 2003).

One explanation for this may be due to the mother still being seen as the primary decision maker for parenting practices. However, with fathers becoming increasingly associated with more active parenting (Dodd & Jackiewicz, 2015), it is important to understand how views of co‐sleeping between both parents are influenced by external judgments, and how this may, in turn, affect other behaviors and cognitions. Germo et al. (2007) found that sleep arrangements are a family matter, with the fathers' attitude influencing overall satisfaction, such as with marital quality. Other research suggests that a strong parental partnership in parenting decisions may be a buffer against the negative effects of criticism (Shimizu & Teti, 2018).

There have been studies that looked specifically at partners' attitudes towards co‐sleeping (Ball et al., 2000; Germo et al., 2007; Gettler et al., 2021), aware of the importance of their inclusions within their research. Other studies, such as Ramos (2003) deliberately examined the attitudes of fathers, finding that mothers generally reported that they shared similar beliefs about co‐sleeping with their partners although these perspectives came from mothers rather than fathers themselves.

While these studies give insight into the attitudes of fathers', and potentially the impact that co‐sleeping can have on other outcomes, such as bonding and parental cohesion, none of these studies examined how the decision to co‐sleep (or not) was made within the dyadic couple, and how external influences may affect their views on co‐sleeping. As the decision to co‐sleep may be influenced by both parents, it is important to explore the attitudes being encountered by both sides and the interplay of these.

### Limitations

This review has several limitations. Firstly, determining the effect of the attitude at times was difficult to extract (e.g., in survey data, associations between behavior and attitudes were noted and extracted, but it is unknown if the attitudes of the source itself (e.g., partners, friends, culture) directly resulted in the behavior change). While within a survey both sides are typically reported, results in the ‘majority’ were typically reported. This may have resulted in some bias in the extraction of survey data in comparison to qualitative data. However, as this is a scoping review, compared to a systematic review, even with this potential bias the overall aim of examining the current literature on co‐sleeping and attitudes encountered from others has still been well examined. Nevertheless, the authors still felt that this information was important to record as part of this review. In addition, as per the inclusion criteria, some quantitative studies that examined the effects of co‐sleeping on other attitudes (e.g., sleep perception, child development) were excluded as the data described associations based on statistical analysis, as opposed to participants themselves identifying and describing the association between encountering an attitude and the effect this had. While the reason for this criterion was included to ensure the explicit experiences as captured by the participants were focused on in this review, it did mean that other studies were excluded that provided insight into associations such as co‐sleeping behavior and sleep perception through statistical methods.

Another limitation revolves around the definition of co‐sleeping. As the search allowed for all definitions of co‐sleeping to be included, there were some studies in which the reported results remained ambiguous, which can limit the findings of this review. While efforts were made to highlight what the extracted data was referring to where possible, and in most cases, results were focused on bed‐sharing, the ambiguity should still be considered.

Finally, only studies that were peer‐reviewed, and primary research were included. As such, an expansion of the data search strategy sources would have likely produced a larger selection of articles, including ethnographic studies published in book chapters and potentially additional results, providing further insights.

## CONCLUSIONS AND RECOMMENDATIONS

This review examined and synthesized the available literature on the attitudes towards parent–child co‐sleeping that people encounter, and the effects of these attitudes. While the scope of the literature was limited, the review did highlight some important points. Primarily, research in this area has been done in Western countries and focuses on the perspectives of caregivers (primarily mothers). Parents/caregivers most commonly encounter encouraging attitudes about co‐sleeping from their extended family members and cultures, while discouraging attitudes come from healthcare professionals. While studies discussed where these attitudes came from, fewer provided information on the effects of these attitudes. The findings suggest that encouraging attitudes may encourage co‐sleeping, whereas discouraging attitudes may discourage the behavior, but the behavior can still occur. The review also highlighted that discouraging attitudes from healthcare professionals can lead to parents avoiding discussions about sleep arrangements with healthcare professionals and potentially cause conflicts with other family members, including partners. The review also highlighted how little research has involved partner's attitudes regarding how the decision to co‐sleeping is made within the dyadic relationship.

Based on these findings further research is needed in several areas related to co‐sleeping in Western culture, most specifically in how external attitudes influence the decision to co‐sleep, as well as other behaviors and cognitions such as engagement with healthcare professionals, family satisfaction, parental self‐efficacy, and overall mental health.

## Supporting information


Tables S1–S3.
