# Supplementary material for: Sources of attitudes towards parent–child co‐sleeping and their effects: A systematic scoping review
Source: Fam Process. 2024 Jun 4;63(4):2484–504. doi: 10.1111/famp.13022 (PMC11659088; doi:10.1111/famp.13022)
Supplement: Supplementary file 1 — Tables S1–S3. [file FAMP-63-2484-s001.doc]

**SUPPORTING INFORMATION**

**Table S1.** List of included articles

**Table S2**. Quality Appraisal

**Table S3.** Summarized results of studies

**Table S1**

List of included articles

Abel, S., Park, J., Tipene-Leach, D., Finau, S., & Lennan, M. (2001). Infant care practices in New Zealand: a cross-cultural qualitative study. *Social Science & Medicine, 53*(9), 1135–1148. https://doi.org/10.1016/s0277-9536(00)00408-1

Abels, M., Bosy, C., & Fredriksen, I. M. (2021). Napping alone in the snow and cuddling with mommy at night: An exploratory, qualitative study of Norwegian beliefs on infant sleep. *Infant Behavior & Development, 65*, 101656. https://doi.org/10.1016/j.infbeh.2021.101656

Bailey, C. (2016). Breastfeeding mothers' experiences of bedsharing: A qualitative study. *Breastfeeding Review: Professional Publication of the Nursing Mothers' Association of Australia, 24*(2), 33–40. https://doi.org/10.3316/informit.268164166573611

Caraballo, M., Shimasaki, S., Johnston, K., Tung, G., Albright, K., & Halbower, A. (2016). Knowledge, attitudes, and risk for Sudden Unexpected Infant Death in children of Adolescent mothers: A qualitative study. *The Journal of Pediatrics, 174*, 78–83. https://doi.org/10.1016/j.jpeds.2016.03.031

Chianese, J., Ploof, D., Trovato, C., & Chang, J. C. (2009). Inner-city caregivers’ perspectives on bed sharing with their infants. *Academic Pediatrics*, *9*(1), 26-32. https://doi.org/10.1016/j.acap.2008.11.005

Crane, D., & Ball, H. L. (2016). A qualitative study in parental perceptions and understanding of SIDS-reduction guidance in a UK bi-cultural urban community. *BMC Pediatrics, 16*(22), 23–23. https://doi.org/10.1186/s12887-016-0560-7

Dodd, J., & Jackiewicz, T. (2015). The negotiations of involved fathers and intensive mothers around shared-bed sleeping with infants (co-sleeping). *Health Sociology Review, 24*(2), 213–225. https://doi.org/10.1080/14461242.2015.1032321

Dosanjh, J.S., & Ghuman, Paul A.S. (1996). The cultural context of child-rearing: A study of Indigenous and British Punjabis. *Early Child Development and Care, 126*(1), 39–55. <https://doi.org/10.1080/0300443961260104>

Feld, H., Ceballos Osorio, J., Bahamonde, M., Young, T., Boada, P., & Rayens, M. K. (2021). Poverty and paternal education associated with infant safe sleep intentions in a peri-urban community in Ecuador. *Global Pediatric Health, 8.* https://doi.org/10.1177/2333794X211044112

Gaydos, L. M., Blake, S. C., Gazmararian, J. A., Woodruff, W., Thompson, W. W., & Dalmida, S. G. (2015). Revisiting safe sleep recommendations for African-American infants: Why current counseling is insufficient. *Maternal and Child Health Journal, 19*(3), 496–503. https://doi.org/10.1007/s10995-014-1530-z

Gustafsson, S., Jacobzon, A., Lindberg, B., & Engström, Å. (2022). Parents’ strategies and advice for creating a positive sleep situation in the family. *Scandinavian Journal of Caring Sciences, 36*(3), 830–838. https://doi.org/10.1111/scs.13020

Hatton, R. E. M., & Gardani, M. (2018). Maternal perceptions of advice on sleep in young children: How, what, and when? *British Journal of Health Psychology, 23*(2), 476–495. https://doi.org/10.1111/bjhp.12300

Hauck, F. R., Signore, C., Fein, S. B., & Raju, T. N. (2008). Infant sleeping arrangements and practices during the first year of life. *Pediatrics, 122* Suppl 2, S113–S120. https://doi.org/10.1542/peds.2008-1315o

Herman, S., Adkins, M., & Moon, R. Y. (2015). Knowledge and beliefs of African-American and American Indian parents and supporters about infant safe sleep. *Journal of Community Health, 40*(1), 12–19. https://doi.org/10.1007/s10900-014-9886-y

Hirai, A. H., Kortsmit, K., Kaplan, L., Reiney, E., Warner, L., Parks, S. E., Perkins, M., Koso-Thomas, M., D'Angelo, D. V., & Shapiro-Mendoza, C. K. (2019). Prevalence and factors associated with safe infant sleep practices. *Pediatrics, 144*(5), e20191286. https://doi.org/10.1542/peds.2019-1286

Hodges, N. L., McKenzie, L. B., Anderson, S. E., & Katz, M. L. (2018). Exploring lactation consultant views on infant safe sleep. *Maternal and Child Health Journal, 22*(8), 1111–1117. https://doi.org/10.1007/s10995-018-2495-0

Hooker, E., Ball, H. L., & Kelly, P.J. (2001). Sleeping like a baby: Attitudes and experiences of bedsharing in Northeast England. *Medical Anthropology, 19*(3), 203–222. https://doi.org/10.1080/01459740.2001.9966176

Jones, H., Barber, C. C., Nikora, L. W., & Middlemiss, W. (2017). Maori child rearing and infant sleep practices. *New Zealand Journal of Psychology, 46*(3), 30-37*.*

Lapps Wert, K. M., Lindemeyer, R., & Spatz, D. L. (2015). Breastfeeding, co-sleeping and dental health advice. *MCN, the American Journal of Maternal Child Nursing, 40*(3), 174–179. https://doi.org/10.1097/NMC.0000000000000129

Liamputtong, P. (2002). Childrearing practices and child health among the Hmong in Australia: Implications for health services. *International Journal of Health Services*, *32*(4), 817–836. https://doi.org/10.2190/TTLQ-YC48-GTVQ-3DJH

MacFarlane, M., Thompson, J. M. D., Mitchell, E. A., Lawton, B., McLardy, E. M., Jonas, S. D., Tepania‐Palmer, G., Roa, T., Warren, G., & Jowsey, T. (2021). Pēpē‐infant sleep practices and sudden unexpected death in infancy in Aotearoa New Zealand. *International Journal of Gynecology and Obstetrics, 155*(2), 305–317. https://doi.org/10.1002/ijgo.13910

Martinez, S. M., & Thompson-Lastad, A. (2015). Latino parents' insight on optimal sleep for their preschool-age child: does context matter? *Academic Pediatrics, 15*(6), 636–643. https://doi.org/10.1016/j.acap.2015.07.003

McKenna, J. J., & Volpe, L. E. (2007). Sleeping with baby: An internet-based sampling of parental experiencers, choices, perceptions, and interpretations in a western industrialised context. *Infant and Child Development, 16*(4), 359-385. https://doi.org/10.1002/icd.525

Morelli, G. A., Rogogg, B., Oppenheim, D., & Goldsmith, D. (1992). Cultural variations in infants’ sleeping arrangements: questions of independence. *Developmental Psychology, 28*(4), 604-613.

Ramos, K. D. (2003). Intentional versus reactive cosleeping. *Sleep Res Online, 5*(4):141– 147.

Sadler, L. S., Banasiak, N., Canapari, C., Crowley, A. A., Fenick, A., O'Connell, M., Ordway, M. R., Sude, L., Trevino, S., & Redeker, N. S. (2020). Perspectives on sleep from multiethnic community parents, pediatric providers, and childcare providers. *Journal of Developmental and Behavioral Pediatrics, 41*(7), 540–549. https://doi.org/10.1097/DBP.0000000000000799

Schaeffer, P., & Asnes, A. G. (2018). What do pediatricians tell parents about bed-sharing? *Maternal and Child Health Journal, 22*(1), 51–58. https://doi.org/10.1007/s10995-017-2353-5

Smith, L. A., Colson, E. R., Rybin, D., Margolis, A., Colton, T., Lister, G. & Corwin, M. J. (2010). Maternal assessment of physician qualification to give advice on AAP-recommended infant sleep practices related to SIDS. *Academic Pediatrics, 10*(6), 383–388. https://doi.org/10.1016/j.acap.2010.08.006

Specker, B. L., Minett, M., Beare, T., Poppinga, N., Carpenter, M., Munger, J., Strasser, K., & Ahrendt, L. (2020). Safe sleep behaviors among South Dakota mothers and the role of the healthcare provider. *South Dakota medicine: the journal of the South Dakota State Medical Association, 73*(4), 152–162.

Stiffler, D., Cullen, D., Stephenson, E., Luna, G., & Hartman, T. D. (2016). When baby stops breathing. *Clinical Nursing Research, 25*(3), 310–324. https://doi.org/10.1177/1054773815619580

Stiffler, D., Matemachani, S. M., & Crane, L. (2020). Considerations in Safe to Sleep® messaging: Learning from African–American mothers. *Journal for Specialists in Pediatric Nursing, 25*(1). https://doi.org/10.1111/jspn.12277

Tan, T. X., Marfo, K., & Dedrick, R. F. (2009). Preschool-age adopted Chinese children's sleep problems and family sleep arrangements. *Infant and Child Development, 18*(5), 422–440. https://doi.org/10.1002/icd.630

Weimer, S. M., Dise, T. L., Evers, P.B., Ortiz, M. A., Welidaregay, W., & Steinmann, W. C. (2002). Prevalence, predictors, and attitudes toward cosleeping in an urban pediatric center. *Clinical Pediatrics, 41*(6), 433–438. https://doi.org/10.1177/000992280204100609

**Table 2**

*Quality Appraisal of Studies*

| **Qualitative Studies** | | | | | | | | | | | | |
| --- | --- | --- | --- | --- | --- | --- | --- | --- | --- | --- | --- | --- |
| **Reference** | **Method** | **Is there congruity between the stated philosophical perspective and the research methodology?** | **Is there congruity between the research methodology and the research question or objectives?** | **Is there congruity between the research methodology and the methods used to collect data?** | **Is there congruity between the research methodology and the representation and analysis of data?** | **Is there congruity between the research methodology and the interpretation of results?** | **Is there a statement locating the researcher culturally or theoretically?** | **Is the influence of the researcher on the research, and vice- versa, addressed?** | **Are participants, and their voices, adequately represented?** | **Is the research ethical according to current criteria or, for recent studies, and is there evidence of ethical approval by an appropriate body?** | **Do the conclusions drawn in the research report flow from the analysis, or interpretation, of the data?** | **JBI Total Quality Score** |
| Abel et al., 2001 | Focus groups and interviews | Yes | Yes | Yes | Yes | Yes | Yes | No | Yes | Yes | Yes | 9/10 |
| Abels et al., 2021* | Mixed Methods; Interview data included (Study 2) | Yes | Yes | Yes | Yes | Yes | No | No | Yes | Yes | Yes | 8/10 |
| Bailey, 2016 | Interviews | Yes | Yes | Yes | Yes | Yes | No | No | Yes | Yes | Yes | 8/10 |
| Caraballo et al., 2016 | Focus groups | Yes | Yes | Yes | Yes | Yes | No | No | Yes | Yes | Yes | 8/10 |
| Chianese et al., 2009 | Focus groups | Yes | Yes | Yes | Yes | Yes | No | No | Yes | Yes | Yes | 8/10 |
| Crane & Ball, 2016 | Interviews | Yes | Yes | Yes | Yes | Yes | Yes | No | Yes | Yes | Yes | 9/10 |
| Dodd & Jackiewicz, 2015 | Interviews and focus groups | Yes | Yes | Yes | Yes | Yes | Yes | Yes | Yes | Yes | Yes | 10/10 |
| Dosanjh & Ghuman, 1996 | Interviews | Yes | Yes | Yes | Unsure | Yes | No | Yes | Yes | No | Yes | 7/10 |
| Gaydos et al., 2015 | Focus groups and interviews | Yes | Yes | Yes | Yes | Yes | No | No | Yes | Yes | Yes | 8/10 |
| Gustafsson et al., 2022 | Survey - open responses | Yes | Yes | Yes | Yes | Yes | No | No | Yes | Yes | Yes | 8/10 |
| Hatton & Gardani, 2018 | Interviews | Yes | Yes | Yes | Yes | Yes | Yes | Yes | Yes | Yes | Yes | 10/10 |
| Herman et al., 2015 | Focus groups | Yes | Yes | Yes | Yes | Yes | No | No | Unclear | Yes | Yes | 7/10 |
| Hodges et al., 2018 | Focus Groups | Yes | Yes | Yes | Yes | Yes | No | No | Yes | Yes | Yes | 8/10 |
| Hooker et al., 2001 | Interviews | Yes | Yes | Yes | Yes | Yes | No | No | Yes | Yes | Yes | 8/10 |
| Jones et al., 2017* | Mixed methods (only interview data extracted) | Yes | Yes | Yes | Yes | Yes | Yes | Yes | Yes | Yes | Yes | 10/10 |
| Lapps Wert et al., 2015 | Interviews | Unclear | Yes | Yes | Yes | Yes | No | No | Yes | Yes | Yes | 7/10 |
| Liamputtong, 2002 | Ethnographic interviews | Yes | Yes | Yes | Yes | Yes | Yes | Yes | Yes | No | Yes | 9/10 |
| MacFarlane et al., 2021 | Interviews | Yes | Yes | Yes | Yes | Yes | Yes | Yes | Yes | Yes | Yes | 10/10 |
| Martinez et al. & Thompson-Lastad, 2015 | Interviews | Yes | Yes | Yes | Yes | Yes | No | No | Yes | Yes | Yes | 8/10 |
| McKenna & Volpe, 2007 | Ethnographic narratives | Yes | Yes | Yes | Yes | Yes | No | No | Yes | Yes | Yes | 8/10 |
| Morelli et al., 1992 | Interviews | Yes | Yes | Yes | Yes | Unclear | No | No | Yes | Unclear | Yes | 6/10 |
| Sadler et al., 2020 | Interviews | Yes | Yes | Yes | Yes | Yes | No | No | Yes | Yes | Yes | 8/10 |
| Schaeffer & Asnes, 2018 | Interviews | Yes | Yes | Yes | Yes | Yes | No | No | Yes | Yes | Yes | 8/10 |
| Stiffler et al., 2016 | Interviews | Yes | No | Yes | Yes | Yes | No | No | Yes | Yes | Yes | 7/10 |
| Stiffler et al., 2020 | Focus Groups | Yes | Yes | Yes | Yes | Yes | No | No | Yes | Yes | Yes | 8/10 |
| **Cross-sectional quantitative studies** | | | | | | | | | | | | |
| **Reference** | **Method** | **Were the criteria for inclusion in the sample clearly defined?** | **Were the study subjects and the setting described in detail?** | **Was the exposure measured in a valid and reliable way?** | **Were objective, standard criteria used for measurement of the condition?** | **Were confounding factors identified?** | **Were strategies to deal with confounding factors stated?** | **Were the outcomes measured in a valid and reliable way?** | **Was appropriate statistical analysis used?** | **-** | **-** | **JBI Total Quality Score** |
| Hauck et al., 2008 | Survey | Yes | Yes | Yes | Yes | Yes | Yes | Yes | Yes | - | - | 8/8 |
| Hirai et al., 2019 | Survey and Maternal Reports | Yes | Yes | Yes | Yes | Yes | Yes | Yes | Yes | - | - | 8/8 |
| Feld et al., 2021** | Mixed Methods; Survey + open ended responses | Yes | Yes | Yes | Yes | Unclear | Unclear | Yes | Yes | - | - | 6/8 |
| Ramos, 2003 | Survey | Yes | Yes | Yes | Yes | Yes | Yes | Yes | Yes | - | - | 8/8 |
| Smith et al., 2010 | Survey | Yes | Yes | Yes | Yes | Yes | Yes | Yes | Yes | - | - | 8/8 |
| Specker et al., 2020 | Survey | Yes | Yes | Unclear | Unclear | Yes | Yes | Unclear | Yes | - | - | 5/8 |
| Tan et al., 2009 | Survey | Yes | Yes | Yes | Yes | Yes | Yes | Yes | Yes | - | - | 8/8 |
| Weimer et al., 2002 | Survey | Yes | Yes | Unclear | Unclear | Yes | Yes | Unclear | Yes | - | - | 5/8 |
| Note. *No JBI appraisal checklist available for mixed methods. Used qualitative checklist as this was the data extracted and used in the review. **No JBI appraisal checklist available for mixed methods. Used cross-sectional checklist as design is quantitatively focused. | | | | | | | | | | | | |

**Table S3**

*Summarized Results of Studies of Parents/Caregivers*

| **Article reference** | **Culture & tradition** | **Parents & extended family** | **Partner** | **Friends** | **Society** | **Healthcare professional** | **Other** | **Categorisation of attitude towards co-sleeping** | **Effects of the attitude reported** | **Categorised effect on participant's *attitude* towards co-sleeping** | **Categorised effect on participant's co-sleeping *behaviour*** | **Effect on other attitudes** | **Effect on other behaviours** |
| --- | --- | --- | --- | --- | --- | --- | --- | --- | --- | --- | --- | --- | --- |
| Abel et al., 2001 | ● |  |  |  |  |  |  | Encouraging: normal and offers protection | Yes | Positive feelings towards co-sleeping | Engaged/ reinforced behaviour | N/A | N/A |
| Abels et al., 2021 |  |  |  |  |  | ● |  | Discouraging: co-sleeping is dangerous/unsafe | Yes | N/A | N/A | Feeling anger, frustration or worry (negative emotions) | N/A |
| Abels et al., 2021 |  |  |  |  |  | ● |  | Encouraging: no specific attitude | No | N/A | N/A | N/A | N/A |
| Abels et al., 2021 |  |  |  |  |  | ● |  | Neutral: co-sleeping is neither good nor bad, or can be both | No | N/A | N/A | N/A | N/A |
| Abels et al., 2021 |  |  |  |  |  |  | ● | Encouraging: co-sleeping is normal and/or traditional | Yes | Reinforced positive feelings towards bedsharing | Engaged/ reinforced behaviour | Feeling of confidence in choice (positive) | N/A |
| Abels et al., 2021 |  | ● |  |  |  |  |  | Discouraging: no specific attitude | No | N/A | N/A | N/A | N/A |
| Bailey, 2016 |  |  |  |  |  | ● |  | Discouraging: co-sleeping is dangerous/unsafe | Yes | Unsure if they should bedshare | Delayed, stopped, avoided or did not engage in behaviour | N/A | N/A |
| Bailey, 2016 |  |  |  |  |  | ● |  | Encouraging: no specific attitude | Yes | Reinforced positive feelings towards bedsharing | Engaged/ reinforced behaviour | N/A | N/A |
| Bailey, 2016 |  |  |  | ● |  |  |  | Encouraging: no specific attitude | Yes | Considered trying bed-sharing to aid with breastfeeding, infant sleep and unsettled baby | Engaged/ reinforced behaviour | N/A | N/A |
| Bailey, 2016 |  | ● |  |  |  |  |  | Encouraging: co-sleeping is normal and/or traditional | Yes | N/A | Engaged/ reinforced behaviour | N/A | Comfort engaging in co-sleeping with others/in front of others |
| Bailey, 2016 |  |  |  | ● |  |  |  | Discouraging: no specific attitude | Yes | N/A | N/A | N/A | Conceal co-sleeping practices |
| Bailey, 2016 |  |  | ● |  |  |  |  | Encouraging: co-sleeping is normal and/or traditional | Yes | N/A | N/A | Feeling of support from others | N/A |
| Bailey, 2016 |  |  |  |  |  | ● |  | Discouraging: co-sleeping is dangerous/unsafe | Yes | N/A | N/A | Feeling judged, under surveillance (negative) | Conceal co-sleeping practices |
| Bailey, 2016 |  |  |  |  |  |  | ● | Discouraging: no specific attitude | Yes | N/A | N/A | Feeling anger, frustration or worry (negative emotions) | N/A |
| Caraballo et al., 2016 |  |  |  |  |  | ● |  | Discouraging: co-sleeping is dangerous/unsafe | Yes | No effect - instinct trumps advice from any source | No effect | N/A | N/A |
| Caraballo et al., 2016 |  | ● |  |  |  |  |  | Discouraging: co-sleeping is an undesirable habit | Yes | No effect - instinct trumps advice from any source | No effect | N/A | N/A |
| Chianese et al., 2009 | ● |  |  |  |  |  |  | Encouraging: co-sleeping is normal and/or traditional | Yes | Co-sleeping is a positive practice | Engaged/ reinforced behaviour | Feeling of confidence in choice (positive) | N/A |
| Chianese et al., 2009 |  | ● |  |  |  |  |  | Encouraging: no specific attitude | No | N/A | N/A | N/A | N/A |
| Chianese et al., 2009 |  |  |  | ● |  |  |  | Encouraging: no specific attitude | No | N/A | N/A | N/A | N/A |
| Chianese et al., 2009 |  | ● |  |  |  |  |  | Discouraging: co-sleeping is an undesirable habit | No | N/A | N/A | N/A | N/A |
| Chianese et al., 2009 |  |  |  | ● |  |  |  | Discouraging: co-sleeping is an undesirable habit | No | N/A | N/A | N/A | N/A |
| Chianese et al., 2009 |  |  |  |  |  | ● |  | Discouraging: co-sleeping is dangerous/unsafe | Yes | No effect - advice ignored | No effect | N/A | N/A |
| Chianese et al., 2009 |  |  |  |  |  | ● |  | Neutral: co-sleeping is neither good nor bad, or can be both | Yes | No effect - but considered strategies | No effect | N/A | N/A |
| Crane & Ball, 2016 | ● |  |  |  |  |  |  | Encouraging: co-sleeping is normal and/or traditional | Yes | Strengthens parent's attitude that bed-sharing is positive | Engaged/ reinforced behaviour | N/A | N/A |
| Dodd & Jackiewicz, 2015 |  |  | ● |  |  |  |  | Discouraging: co-sleeping is an undesirable habit | Yes | N/A | Delayed, stopped, avoided or did not engage in behaviour | N/A | Confrontations with other people about co-sleeping |
| Dodd & Jackiewicz, 2015 |  |  | ● |  |  |  |  | Discouraging: co-sleeping is an undesirable habit | Yes | N/A | Delayed, stopped, avoided or did not engage in behaviour | N/A | Confrontations with other people about co-sleeping |
| Dodd & Jackiewicz, 2015 |  |  | ● |  |  |  |  | Encouraging: co-sleeping offers protection, bonding/love and/or has practical benefits | Yes | N/A | N/A | N/A | Confrontations with other people about co-sleeping |
| Dodd & Jackiewicz, 2015 | ● |  |  |  |  |  |  | Encouraging: normal and offers protection | Yes | Reinforced positive feelings towards bedsharing | Engaged/ reinforced behaviour | N/A | N/A |
| Dosanjh & Ghuman, 1996 | ● |  |  |  |  |  |  | Encouraging: co-sleeping offers protection, bonding/love and/or has practical benefits | Yes | Positive feelings towards co-sleeping | Engaged/ reinforced behaviour | N/A | N/A |
| Feld et al., 2021 | ● |  |  |  |  |  |  | Encouraging: co-sleeping is normal and/or traditional | Yes | N/A | Engaged/ reinforced behaviour | N/A | N/A |
| Gustafsson et al., 2022 |  |  |  |  |  | ● |  | Discouraging: no specific attitude | No | N/A | N/A | N/A | N/A |
| Hatton & Gardani, 2018 |  |  |  |  |  | ● |  | Discouraging: co-sleeping is dangerous/unsafe | Yes | N/A | N/A | N/A | Conceal co-sleeping practices |
| Hauck et al., 2008 |  |  |  |  |  | ● |  | Encouraging: no specific attitude | No | N/A | N/A | N/A | N/A |
| Hauck et al., 2008 |  |  |  |  |  | ● |  | Discouraging: no specific attitude | Yes | N/A | Delayed, stopped, avoided or did not engage in behaviour | N/A | N/A |
| Hauck et al., 2008 |  | ● |  |  |  |  |  | Discouraging: co-sleeping is not the norm | Yes | N/A | Delayed, stopped, avoided or did not engage in behaviour | N/A | N/A |
| Herman et al., 2015 |  |  | ● |  |  |  |  | Discouraging: no specific attitude | Yes | N/A | Delayed, stopped, avoided or did not engage in behaviour | N/A | N/A |
| Hirai et al., 2019 |  |  |  |  |  | ● |  | Discouraging bed-sharing/ encouraging room-sharing: no specific attitude | Yes | N/A | Delayed, stopped, avoided or did not engage in bed-sharing behaviour/ encouraged/reinforced room-sharing behaviour | N/A | N/A |
| Hooker et al., 2001 |  | ● |  |  |  |  |  | Encouraging: no specific attitude | Yes | N/A | No effect | N/A | N/A |
| Hooker et al., 2001 |  |  |  | ● |  |  |  | Discouraging: co-sleeping is an undesirable habit | Yes | N/A | Delayed, stopped, avoided or did not engage in behaviour | N/A | N/A |
| Hooker et al., 2001 |  |  |  |  |  | ● |  | Discouraging: co-sleeping is dangerous/unsafe | Yes | N/A | N/A | Feeling judged, under surveillance (negative) | Conceal co-sleeping practices |
| Jones et al., 2017 |  | ● |  |  |  |  |  | Encouraging: co-sleeping is normal and/or traditional | Yes | Co-sleeping is a positive practice | Engaged/ reinforced behaviour | N/A | N/A |
| Jones et al., 2017 |  | ● |  |  |  |  |  | Encouraging: co-sleeping offers protection, bonding/love and/or has practical benefits | Yes | N/A | N/A | Feeling judged, under surveillance (negative) | N/A |
| Jones et al., 2017 | ● |  |  |  |  |  |  | Encouraging: co-sleeping offers protection, bonding/love and/or has practical benefits | Yes | N/A | Engaged/ reinforced behaviour | N/A | N/A |
| Lapps Wert et al., 2015 |  |  |  |  |  | ● |  | Discouraging: no specific attitude | Yes | N/A | N/A | N/A | Conceal co-sleeping practices |
| Lapps Wert et al., 2015 |  |  |  |  |  | ● |  | Encouraging: no specific attitude | No | N/A | N/A | N/A | N/A |
| Liamputtong, 2002 | ● |  |  |  |  |  |  | Encouraging: co-sleeping offers protection, bonding/love and/or has practical benefits | Yes | Positive feelings towards co-sleeping | Engaged/ reinforced behaviour | N/A | N/A |
| Liamputtong, 2002 | ● |  |  |  |  |  |  | Encouraging: co-sleeping offers protection, bonding/love and/or has practical benefits | Yes | Positive feelings towards co-sleeping | Engaged/ reinforced behaviour | N/A | N/A |
| Liamputtong, 2002 | ● |  |  |  |  |  |  | Encouraging: co-sleeping offers protection, bonding/love and/or has practical benefits | Yes | Positive feelings towards co-sleeping | Engaged/ reinforced behaviour | N/A | N/A |
| Liamputtong, 2002 |  |  |  |  |  | ● |  | Discouraging: co-sleeping is dangerous/unsafe | Yes | No effect - advice ignored | No effect | N/A | Conceal co-sleeping practices |
| MacFarlane et al., 2021 | ● |  |  |  |  |  |  | Encouraging: co-sleeping is normal and/or traditional | Yes | Motivated to co-sleep | N/A | N/A | N/A |
| MacFarlane et al., 2021 |  |  |  |  |  | ● |  | Discouraging: co-sleeping is dangerous/unsafe | Yes | Concerns about co-sleeping | Delayed, stopped, avoided or did not engage in behaviour | N/A | N/A |
| MacFarlane et al., 2021 |  | ● |  |  |  |  |  | Encouraging: co-sleeping is normal and/or traditional | Yes | Motivated to co-sleep | N/A | N/A | N/A |
| Martinex et al., 2015 | ● |  |  |  |  |  |  | Encouraging: co-sleeping is normal and/or traditional | No | N/A | N/A | N/A | N/A |
| McKenna & Volpe, 2007 |  |  |  |  |  | ● |  | Discouraging: co-sleeping is an undesirable habit | No | N/A | N/A | N/A | N/A |
| Morelli et al., 1992 |  |  |  | ● |  |  |  | Discouraging: co-sleeping is an undesirable habit | Yes | Co-sleeping should be avoided because it is a habit that is difficult to break | Delayed, stopped, avoided or did not engage in behaviour | N/A | N/A |
| Ramos, 2003 |  |  | ● |  |  |  |  | Encouraging: no specific attitude | Yes | N/A | N/A | Feeling of support from others | N/A |
| Ramos, 2003 |  | ● |  |  |  |  |  | Discouraging: no specific attitude | No | N/A | N/A | N/A | N/A |
| Ramos, 2003 |  |  |  |  | ● |  |  | Discouraging: no specific attitude | No | N/A | N/A | N/A | N/A |
| Ramos, 2003 |  |  |  | ● |  |  |  | Encouraging: no specific attitude | No | N/A | N/A | N/A | N/A |
| Ramos, 2003 |  |  |  | ● |  |  |  | Discouraging: no specific attitude | No | N/A | N/A | N/A | N/A |
| Ramos, 2003 |  |  | ● |  |  |  |  | Encouraging: no specific attitude | Yes | N/A | N/A | Feeling of support from others | N/A |
| Ramos, 2003 |  | ● |  |  |  |  |  | Discouraging: no specific attitude | No | N/A | N/A | N/A | N/A |
| Ramos, 2003 |  |  |  |  | ● |  |  | Discouraging: no specific attitude | No | N/A | N/A | N/A | N/A |
| Ramos, 2003 |  |  |  | ● |  |  |  | Encouraging: no specific attitude | No | N/A | N/A | N/A | N/A |
| Ramos, 2003 |  |  |  | ● |  |  |  | Discouraging: no specific attitude | No | N/A | N/A | N/A | N/A |
| Ramos, 2003 |  |  | ● |  |  |  |  | Encouraging: no specific attitude | Yes | N/A | N/A | Feeling of support from others | N/A |
| Ramos, 2003 |  | ● |  |  |  |  |  | Discouraging: no specific attitude | No | N/A | N/A | N/A | N/A |
| Ramos, 2003 |  |  |  |  | ● |  |  | Discouraging: no specific attitude | No | N/A | N/A | N/A | N/A |
| Ramos, 2003 |  |  |  | ● |  |  |  | Encouraging: no specific attitude | No | N/A | N/A | N/A | N/A |
| Ramos, 2003 |  |  |  | ● |  |  |  | Neutral: co-sleeping is neither good nor bad, or can be both | No | N/A | N/A | N/A | N/A |
| Ramos, 2003 |  |  | ● |  |  |  |  | Encouraging: no specific attitude | Yes | N/A | N/A | Feeling of support from others | N/A |
| Ramos, 2003 |  | ● |  |  |  |  |  | Discouraging: no specific attitude | No | N/A | N/A | N/A | N/A |
| Ramos, 2003 |  |  |  |  | ● |  |  | Discouraging: no specific attitude | No | N/A | N/A | N/A | N/A |
| Ramos, 2003 |  |  |  | ● |  |  |  | Encouraging: no specific attitude | No | N/A | N/A | N/A | N/A |
| Ramos, 2003 |  |  |  | ● |  |  |  | Neutral: co-sleeping is neither good nor bad, or can be both | No | N/A | N/A | N/A | N/A |
| Sadler et al., 2020 |  |  |  |  |  | ● |  | Discouraging: co-sleeping is dangerous/unsafe | Yes | Fear of co-sleeping | Engaged/ reinforced behaviour | N/A | N/A |
| Sadler et al., 2020 | ● |  |  |  |  |  |  | Encouraging: co-sleeping is normal and/or traditional | Yes | N/A | Engaged/ reinforced behaviour | N/A | N/A |
| Smith et al., 2010 |  |  |  |  |  | ● |  | Discouraging: co-sleeping is dangerous/unsafe | Yes | N/A | N/A | Increased trust in source | N/A |
| Smith et al., 2010 |  |  |  |  |  | ● |  | Neutral: co-sleeping is neither good nor bad, or can be both | Yes | N/A | N/A | Increased trust in source | N/A |
| Specker et al., 2020 |  |  |  |  |  | ● |  | Discouraging bed-sharing: no specific attitude | Yes | N/A | Delayed, stopped, avoided or did not engage in behaviour | N/A | N/A |
| Specker et al., 2020 |  |  |  |  |  | ● |  | Encouraging room-sharing: no specific attitude | Yes | N/A | Engaged/ reinforced behaviour | N/A | N/A |
| Stiffler et al., 2016 |  |  |  |  |  | ● |  | Discouraging: no specific attitude | No | N/A | N/A | N/A | N/A |
| Stiffler et al., 2016 |  |  |  |  |  | ● |  | Neutral: co-sleeping is neither good nor bad, or can be both | No | N/A | N/A | N/A | N/A |
| Stiffler et al., 2020 |  | ● |  |  |  |  |  | Encouraging: no specific attitude | Yes | N/A | Engaged/ reinforced behaviour | N/A | Comfort engaging in co-sleeping with others/in front of others |
| Stiffler et al., 2020 |  |  |  |  |  | ● |  | Discouraging: co-sleeping is dangerous/unsafe | Yes | No change on attitude towards co-sleeping | No effect | Feeling judged, under surveillance (negative) | Conceal co-sleeping practices |
| Stiffler et al., 2020 |  |  |  |  |  | ● |  | Discouraging: co-sleeping is dangerous/unsafe | Yes | Negative feeling towards co-sleeping | Delayed, stopped, avoided or did not engage in behaviour | Feeling anger, frustration or worry (negative emotions) | Confrontations with other people about co-sleeping |
| Stiffler et al., 2020 |  |  |  |  |  | ● |  | Discouraging: co-sleeping is dangerous/unsafe | Yes | Negative feeling towards co-sleeping | No effect | Feeling anger, frustration or worry (negative emotions) | N/A |
| Tan et al., 2009 |  |  |  |  |  | ● |  | Discouraging: no specific attitude | No | N/A | N/A | N/A | N/A |
| Tan et al., 2009 |  | ● |  |  |  |  |  | Encouraging: no specific attitude | No | N/A | N/A | N/A | N/A |
| Tan et al., 2009 |  |  |  | ● |  |  |  | Encouraging: no specific attitude | No | N/A | N/A | N/A | N/A |
| Weimer et al., 2002 |  | ● |  |  |  |  |  | Encouraging: no specific attitude | Yes | N/A | Engaged/ reinforced behaviour | N/A | N/A |
